# Optimizing the Management of Cadmium Bioremediation Capacity of Metal-Resistant *Pseudomonas* sp. Strain Al-Dhabi-126 Isolated from the Industrial City of Saudi Arabian Environment

**DOI:** 10.3390/ijerph16234788

**Published:** 2019-11-29

**Authors:** Naif Abdullah Al-Dhabi, Galal Ali Esmail, Abdul-Kareem Mohammed Ghilan, Mariadhas Valan Arasu

**Affiliations:** Addiriyah Chair for Environmental Studies, Department of Botany and Microbiology, College of Science, King Saud University, P.O. Box 2455, Riyadh 11451, Saudi Arabia; gesmail@ksu.edu.sa (G.A.E.); 436107839@student.ksu.edu.sa (A.-K.M.G.); mvalanarasu@ksu.edu.sa (M.V.A.)

**Keywords:** cadmium, biosorption, heavy metal, metal resistant, bacteria bioremediation

## Abstract

In this study, 23 bacterial strains were isolated from a Cadmium (Cd) contaminated soil in the industrial city, Riyadh of Saudi Arabia. Among these isolates six strains were found to withstand cadmium contamination and grow well. From the six isolates *Pseudomonas* sp. strain Al-Dhabi-122–127 were found to resist cadmium toxicity to a higher level. The isolates were subjected to biochemical and 16S rDNA gene sequence characterization to confirm their identification. The bacterial strain Al-Dhabi-124 showed 1.5 times higher Cd-degrading activity than Al-Dhabi-122 and Al-Dhabi-123, and Al-Dhabi-126 exhibited 3.5 times higher Cd-degrading activity, higher than the other strains. An atomic absorption spectrophotometer study showed that the strain Al-Dhabi-126 absorbed Cd, and that the bacterial strain Al-Dhabi-126 was found to tolerate cadmium level up to 2100 µg/mL. The bacterial strain Al-Dhabi-126 showed a maximum Cd removal efficacy at pH between 6.0 and 8.0. The efficacy decreased sharply after an increase in pH (9.0). An optimum temperature of 50 °C and pH 6.0 were found to be effective for the Cd removal process by the isolate. The study indicated that the bacterial strain Al-Dhabi-126 can be used effectively for the bioremediation of heavy metals like cadmium, a major toxic pollutant in industrial effluents.

## 1. Introduction

The bioaccumulation of various heavy metals in the natural environment is a concern to the health of all living organisms. Water pollution caused by industrial effluents carrying heavy metals, toxic sludge, and various solvents affects the quality of water and its dependents. Heavy metals from the industrial wastes entering aquatic ecosystems cause health hazards to animals, plants, humans, and aquatic biotopes [1]. The heavy metals, namely, mercury(Hg), copper(Cu), chromium (Cr), zinc (Zn), cadmium (Cd), and lead (Pb) are mutagenic, toxic to the cells, and induce carcinogenic changes in human beings and other organisms. Also, untreated or partially treated industrial wastewater discharged with heavy metals into water bodies may critically affect the groundwater as well. Microbes have the potential to remediate heavy metal pollutants in the environment. The microbes synthesize various metabolites to degrade the complex wastes and also develop the ability to survive in the presence of various toxic heavy metals in their environment [2]. 

Cadmium (Cd) is one of the major pollutants, and highly toxic to organisms even at very low concentrations. Cd is mainly used in various industries including paint, copper alloy, pulp and paper, mining, alkaline batteries, zinc refining, and fertilizer. Cd enters into animal and human bodies through the food web and bioaccumulate, and may cause various serious diseases [3]. Cd is not required for any biological function but inhibits the DNA-mediated transformation in microorganisms, their cellular enzyme functions, and affects the symbiotic relationship between plants and microbes [4]. Also, the bioaccumulation of Cd in most of the plants may disturb various biochemical functions, structural changes, and physiological processes, including alteration in mineral uptake, photosynthesis function, interfering with the enzymes involved in Calvin cycle and metabolism of carbohydrates, lowers the productivity of crops, and alters antioxidant metabolism in plants [5].

Many methods are applied to eliminate heavy metals from the aquatic environment. The common methods include chemical oxidation, chemical precipitation, reduction, filtration, electrochemical treatment, and extraction using solvents [6]. These traditional methods have various drawbacks including the unpredictable removal of heavy metals and the huge amount of generation of sludge which is highly toxic. The heavy metal removal by means of bioremediation is an alternate way to apply recombinant and naturally available indigenous microorganisms for the effective removal of toxic substances [7]. Bioremediation is environmentally friendly and is cheaper than chemical methods. Also, the dead biomass of bacteria or living microbes is used for the removal of metals through the bioaccumulation and biosorption process [8]. The bioaccumulation process needs energy and it is an oxygen-dependent process. However, biosorption is an independent revisable process and does not require respiration/energy [9]. The important advantage of this process is a high sorption ability, very low operating cost, potent bio sorbent revival, and the possibility of metal recovery [10].

Gram-negative bacteria are capable of resisting and accumulate Cd from the contaminated sites. The biomass of the *P*. *aeruginosa* strain was reported to be highly efficient for the recovery and removal of Cd, Pb, and Cu from a polluted aquatic environment [11]. Also, the dead cells of *P*. *aeruginosa* (dead cell biomass) have the potential for adsorption of Pb and Cd from the aquatic environment polluted with heavy metals [12]. The Cd resistant *Pseudomonas* sp. is capable of biosorbing heavy metals, namely, Ni, Cr, Pb, and Cd. The Cd resistant *P*. *aeruginosa* E1 has a higher potential for biosorption of Cd than dead biomass [13]. The freeze-dried *P*. *aeruginosa* PAO1 cells were found to adsorb Cd from water at an acidic pH value (pH 5.0–6.0) [14]. In a study, *P*. *aeruginosa* KUCd1 has been reported to remove more than 75% Cd within 60 min from the medium during the active growth periods [15]. Also, the genetically engineered *P*. *aeruginosa* effectively removed Cd [16]. It was also reported that the strains from the genus *Pseudomonas* have the potential to remove Cd because of its good biosorption efficacy [17]. Most of the microorganisms use many strategies to counter the heavy metal stress which include active efflux of metals; metal ions sequestration, Cd accumulation, and enzymatic detoxification [18,19]. 

In the present investigation, a potent Cd resistant strain Al-Dhabi 126 was isolated from industrial effluents with Cd pollutants in Saudi Arabia. It was identified as *Pseudomonas* sp. based on a biochemical characterization and 16S rDNA sequence analysis. Cadmium tolerance level and the optimum conditions for its removal through biosorption were evaluated. Also, Cd removal efficacy of the isolated *Pseudomonas* sp. Al-Dhabi-126 promises a new hope for employing it as a Cd bio remediating agent.

## 2. Materials and Methods

### 2.1. Sample Collection

The wastewater sample was collected from three sites (Al-hair, Al-batha wally, and Al-mansouriyah) in Riyadh, where industrial effluents are discharged. The sample was collected in the labeled containers and kept in ice (2–8 °C) and transported to the laboratory for the analysis of heavy metal degrading bacteria. 

### 2.2. Primary Screening of Cd Resistant Bacteria

To find out heavy metal resistant bacteria from the samples, 150 µg/L of Cd was added to Luria Bertani (LB) agar plates (g/L) (peptone 10.0, yeast extract 5.0, sodium chloride 5.0, dextrose anhydrate 10.0 and agar 30.0 and pH 7.0) and incubated for one week at 37 °C. For every 24 h the colony morphology and numbers of bacterial colonies formed were observed. After preliminary observation, the samples showing Cd-degrading isolates were serially diluted by standard method. To the control plates, Cd was not incorporated. The growth of bacteria was monitored continuously. From the colonies with various morphological and growth potentials, rapid growing heavy metal tolerant bacteria were selected and purified for further analysis [13].

### 2.3. Evaluation of Minimum Inhibitory Concentration

To analyze minimum inhibitory concentration (MIC) of Cd resistant bacteria, the selected strains were grown on Cd-incorporated LB agar medium with a dose level 50 µg/mL–2200 µg/mL [9]. Initially, the concentration of the Cd was 50 µg/mL and the strain growing on the final Cd concentration (2200 µg/mL) was further tested and the MIC dose was the concentration at which the bacterial strains failed to grow beyond that Cd level that is visible in the Petri plate. MIC was calculated by the method of European food safety authority (EFSA), Parma, Italy, 2012.

### 2.4. Cadmium Removal Assay

The isolated bacterial strains were cultured in the Luria Bertani (LB) broth containing Cd (500 µg/mL). The culture was incubated on a rotary shaker at 37 °C for 24 h. After every 4 h, the culture was centrifuged (10,000 rpm, 10 min). To analyze the effect of temperature, the isolates were grown at various temperatures (10–60 °C) and to evaluate the influence of different pH the culture was grown at various pH values (2.0–12.0). The culture medium without Cd was used as the negative control. The centrifuged cell-free supernatant was stored at 4 °C for the analysis of Cd remediation. The Cd content of the cell-free supernatant was detected using a GBC932 atomic absorption spectrometry with a Cd hollow cathode lamp at 228.8 nm. Also, the optical density of the bacterial isolates was registered individually to monitor the growth rate of bacteria in relation to Cd removing efficacy [3]. 

### 2.5. Identification of Bacteria

The bacterial isolate that survived in a high dose of Cd was isolated and identified using morphological and biochemical tests such as, Gram-staining, oxidase, citrate, arabinose, cellobiose, raffinose, xylose, Voges–Proskauer, and lactose hydrolysis [20]. Then the selected bacterial isolate was further subjected to 16S rDNA sequencing for identification confirmation. The genomic DNA of the selected strain was isolated by Triton–Prep method. The 16S rDNA gene of the selected strain was purified, amplified using bacterial universal forward and reverse primer (27F - 5′AGAGTTTGATCMTGGCTCAG3′ and 1492R 5′ 5′TACGGYTACCTTGTTACGACTT3′). The polymerization reaction was performed by initial denaturation (5 min) (94 °C), denaturation (94 °C) for 35 cycles, annealing (30 s, 52 °C), and elongation (40 s, 72 °C). The final extension was performed for 10 s at 72 °C. The amplified DNA was loaded on the agarose gel and separated the DNA using TBE buffer (Tris 89 mM, EDTA 2 mM, Boric acid 89 mM, and pH = 7). Further, the purified product was subjected to partial 16S rDNA sequencing analysis and a similar sequence was compared. 

## 3. Results and Discussion

### 3.1. Screening of Cd Resistant Bacteria

**Figure 1:**
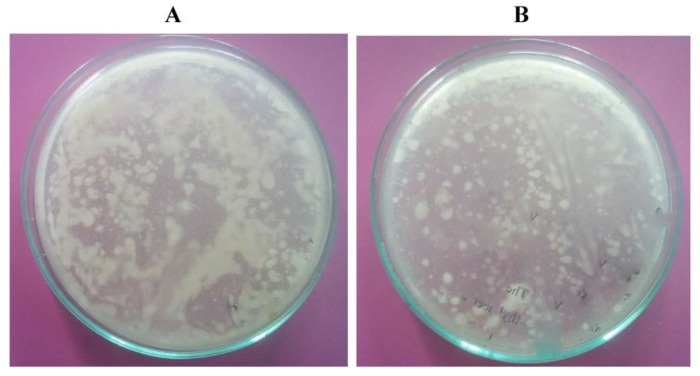
Growth of bacteria on a Luria Bertani (LB) agar medium (**A**) control plate (without cadmium) (**B**) 500 µg/mL cadmium incorporated in an LB agar. The sample was serially diluted and loaded onto the LB agar and incubated at 37 °C for one week.

The absorbance of heavy metals by bacterial biomass is an advanced bioengineering tool for the effective removal of various metal contaminants. In the current study, Cd biodegrading bacterial strains were isolated from the metal contaminated soil (Table 1). The isolated bacteria varied significantly in their effectiveness at absorbing Cd from the aqueous medium. Many factors including, pH, temperature, and C/N ratio are reported to influence biosorption. The ability to degrade heavy metals has been reported in *Enterobacter* spp., *Flavobacterium* spp., *Pseudomonas* spp., *Bacillus* spp., and *Micrococcus* spp. and the bioremediation ability varied among different strains. In bacteria, the absorption ability was found to be higher due to high surface-to-volume ratios and the presence of active chemo sorption sites on cell wall surfaces [21]. However, the metal absorption ability of these bacterial isolates depends on its tolerance limits. Both Gram-negative and Gram-positive bacteria are reported to show heavy metal tolerance. The bacterial species such as *Alcaligenes xylosoxidans, Klebsiella planticola, Pseudomonas fluorescens, Comamonas testosteroni, Pseudomonas putida*, *Serratia liquefaciens,* and *Pseudomonas* sp. showed resistance to Cd between 3 and 11 mM [22]. In this study, bacteria showing Cd resistance was observed when the LB agar plate was supplemented with Cd (500 µg/mL). It indicated that the bacterial isolates can grow by effectively degrading Cd. In the present study, the control plate showed 13.2 × 10^2^ bacterial colonies, where 32.5% of isolates were Cd resistant (Figure 1). A total of 23 bacterial strains were isolated from the sample, among these, six bacterial strains were selected for further studies based on their capacity to grow rapidly on the LB agar containing Cd. The growth from previous investigations revealed that the microorganisms such as *Thiobacillus ferrooxidans*, *Citrobacter* sp., *Bacillus cereus*, *Bacillus subtilis*, *Saccharomyces cerevisiae*, *Micrococcus luteus*, *Pseudomonas aeruginosa*, *Aspergillus flavus*, *Rhizopus arrhizus,* and *Acinetobacter baumannii* are good to remove heavy metals from contaminated soil [23]. 

Heavy metals tolerant bacterial isolates from the natural environment play a significant role in bioremediation of contaminated soil. Sulaimon et al. [24] isolated 12 bacterial strains capable of degrading the heavy metals such as zinc, lead, mercury, and copper from an industrial area for bioremediation. Alternatively, Day and Paul [25] isolated six arsenic, lead, and Cd resistant strains from the metal-contaminated environment. It was reported that *Ochrobactrum* sp. reduces lead, chromium, zinc, Cd, cobalt, copper, and nickel in various experimental trials [26]. Naik et al. [27] isolated and characterized hexavalent chromium degrading *Bacillus cereus* IST105 from electroplating effluent. 

### 3.2. Relative Growth of Bacteria on LB Medium at Various Concentrations of Cd

**Figure 2:**
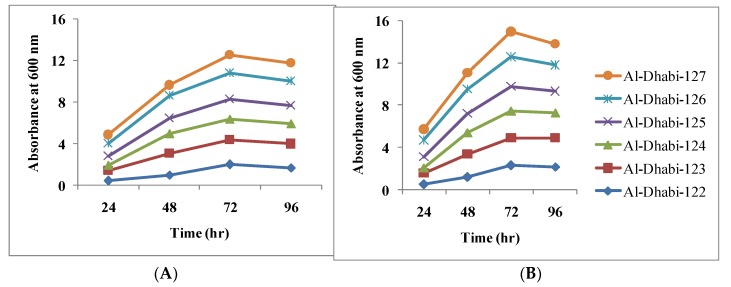
Growth of cadmium resistant bacterial isolates at various incubation times. (**A**) without cadmium (**B**) with 300 ppm cadmium.

Many research works have been performed for the isolation of new heavy metal tolerant bacterial strains for the past three decades. In this study, initially, 150 µg/mL Cd was incorporated with the LB broth with six bacterial isolates and bacterial growth in the medium with or without Cd was recorded. Later, Cd concentration was increased to 2200 µg/mL and the bacterial strains were incubated for 24 h and bacterial growth (OD at 600 nm) was measured using a UV-visible spectrophotometer. In the observation, two bacterial strains showed less biomass at lower Cd concentration (100 µg/mL and 200 µg/mL) and growth was found to be higher at 300 µg/mL concentration of Cd. The growth of the selected bacterial isolate with 300 cadmium and control (without cadmium) was described in Figure 2. Sophia et al. [28] studied the application of Cd resistant strains which were isolated from various environments highly contaminated by heavy metals, including, Cd. 

### 3.3. Bacterial Strains and Colony Morphology 

Among the isolates, 73% of the bacterial strains were circular shaped and 27% bacterial strains were an irregular shape (Table 2). About 28% of strains were flat, 47% were convex, and 26% of the isolates were umbonate. Bacterial margins analysis documented that approximately 9% were irregular, 19% were lobate, and the remaining 72% were entire. The isolated strains also showed various colors in their appearances. About 6% were yellow, orange (7%), brown (7%), red (10%), white (52%), and 18% were white-cream.

The isolated bacterial strains were shaped like a rod. The cellular arrangements of the bacterial strains were scattered (84%) and single (18%). The isolated bacterial strains were both Gram-negative (23%) and Gram-positive (77%). The isolates that thrived well in the Cd-incorporated medium were identified to be Al-Dhabi-122, Al-Dhabi-123, Al-Dhabi-124, Al-Dhabi-125, and Al-Dhabi-126. Al-Dhabi-124 showed 1.5 times higher Cd-degrading activity than Al-Dhabi-122 and Al-Dhabi-123, respectively. Results indicated that the selected microbial species can absorb Cd from the medium by binding the outer membrane with metals. The potent strain, Al-Dhabi-126 was identified as *Pseudomonas* species and 16S rDNA sequence was deposited in GenBank under the accession numberMN709219. 

### 3.4. Bioremediation of Cd by the Bacterial Isolates

**Figure 3:**
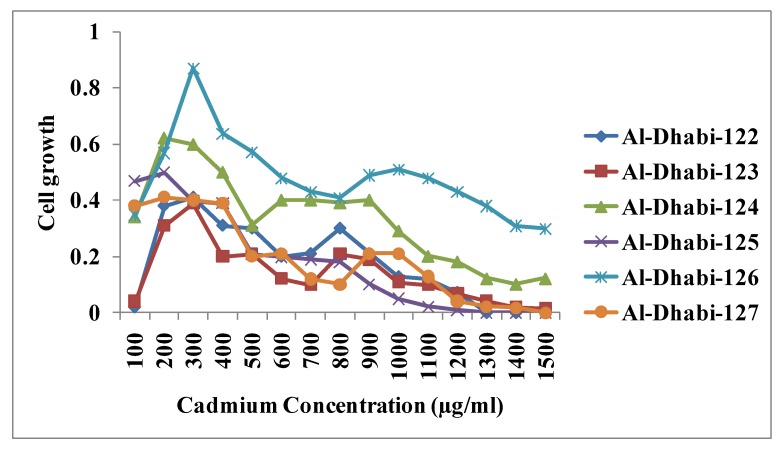
Growth of cadmium degrading bacterial strains at various cadmium concentrations. Optical density was measured after 12 h of incubation at 30 ± 2 °C.

To analyze total Cd biodegradation ability, the control and experimental samples were assayed by the atomic absorption spectrophotometer and the result was compared with the control sample. Among the bacterial strains (Al-Dhabi-122–Al-Dhabi-127), Al-Dhabi-124 showed 1.5 times higher Cd-degrading activity than Al-Dhabi-122 and Al-Dhabi-123 respectively. However, Al-Dhabi-126 showed 3.5 times Cd-degrading activity higher than all the other strains (Figure 3). Results indicated that the selected bacterial isolates can absorb Cd from the medium by binding the outer membrane with metals. It was predicted that the mucous layer of the bacterial cell wall easily interacts with heavy metals by adsorption or absorption. Also, the functional group of the cell wall of bacteria is mostly negatively charged and most of the heavy metals are positively charged, which results in the interaction with microbial cells or through microbial cell membranes. Many bacterial species including *Pseudomonas* [29], *Bacillus* [30], and *Streptomyces* [31] have the significant ability to degrade heavy metals by adsorption or absorption.

**Figure 4:**
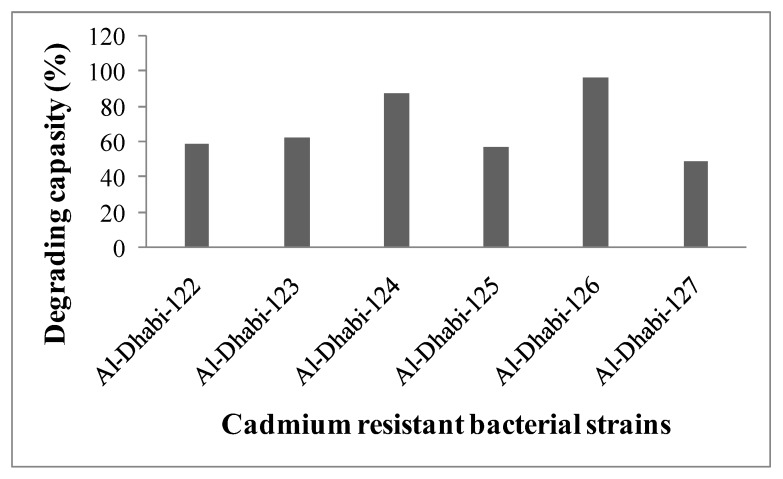
Heavy metal degradation capacity (%) of selected bacterial strains. Each experiment was performed in triplicate analysis and the average value was reported. Total cadmium content was assayed by atomic absorption spectrophotometer under standard assay condition.

**Figure 5:**
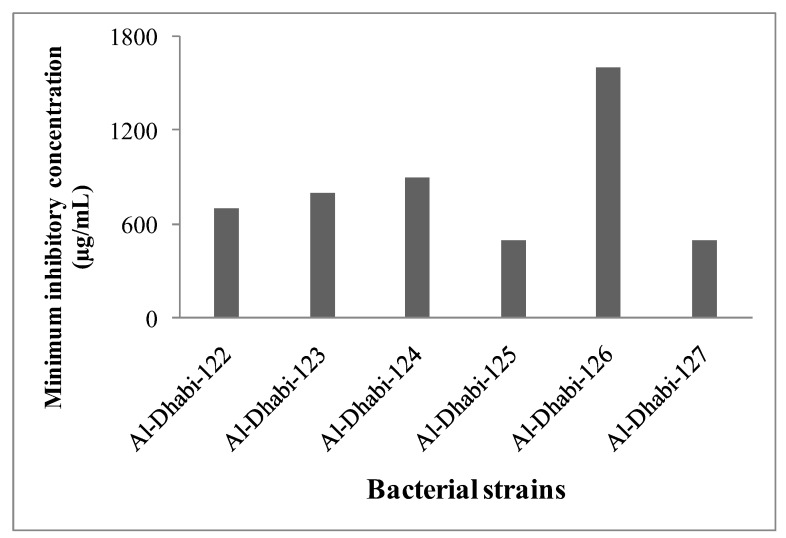
Minimal inhibitory concentration (MIC) of bacterial isolates isolated from heavy metal contaminated sites. Each experiment was performed in triplicate analysis and the average value was reported. Total cadmium content was assayed by atomic absorption spectrophotometer under standard assay condition.

The present investigation revealed the ability of the bacterial strains from the collected samples tolerated Cd in the culture media (Figure 4). The selected bacterial strains showed a Cd tolerance level up to 2100 µg/mL. It was previously reported that bacteria such as *Proteus vulgaris*, *Acinetobacter,* and *Pseudomonas aeruginosa* degrade Chromium, Cadmium, and Lead [32]. Minimum inhibitory concentration (MIC) is the extreme lowest concentration of Cd at which the selected strain growth was completely suppressed. In this study, the MIC of the strain Al-Dhabi-126 was found to be higher than the other five strains. In a study, Yamina et al. [33] reported bacteria, including *Micrococcus luteus* showing resistance to heavy metals such as Cr, Cd, Zn, and Pb. In this study the MIC value of Cd for the strain Al-Dhabi-126 was 2100 µg/mL (Figure 5). Samanta et al. [34] isolated and screened heavy metal resistant bacteria from wastewater. The MIC values of bacterial isolates from wastewater against chromium, zinc, Cd, and lead were between 100 and 2100 μg/mL. The bacterial strain *Micrococcus luteus* reported to show the maximum tolerance (MTC) value for Cr, Ni, and Cd [27,35]. In this study, we have found that the bacterial strains Al-Dhabi-122–126 are capable of absorbing heavy metals from the medium. Among them, strain Al-Dhabi-126 is relatively more efficient than other strains in absorbing Cd.

Heavy metals like Cd and Cr are nonessential elements, have no significant biological role, and are highly toxic to the environment [36]. Many investigations have proven that metal ions at high levels affect the metabolic process of bacteria, their function, growth of the bacterial cells, and bacterial diversity [37].

### 3.5. Effect of Temperature on Cd Removal by Bacterial Strain

**Figure 6:**
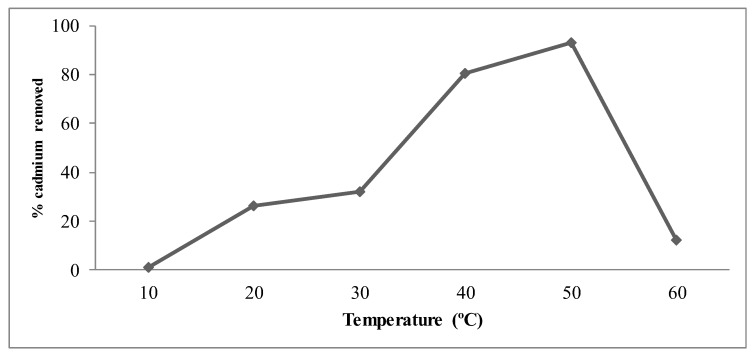
The effect of temperature on cadmium removal efficiency by *Pseudomonas* sp. strain- Al-Dhabi-126.

Temperature is one of the critical factors that affect the growth and metabolism of bacteria. The influence of incubation temperature on Cd removal from the medium is shown in Figure 6. The present findings suggested that the selected bacterial isolate Al-Dhabi-126 preferred an optimum incubation temperature of 40–50 °C for Cd removal and metabolism of bacteria. Higher temperatures above 60 °C did not show any significant Cd removal activity by the selected bacterial strains. This is mainly due to the decrease in metabolic processes at higher temperatures. High incubation temperatures of heavy metal can also denature or inhibit enzyme activity and heavily affect the structure of the plasma membrane and bacterial growth. At low incubation temperatures, Cd removal efficacy by the bacteria was reduced due to the inactivation of enzymes at lower temperatures and the rate of metabolism got decreased considerably.

### 3.6. Effect of pH on Cd Removal by Pseudomonas sp. Al-Dhabi 126

**Figure 7:**
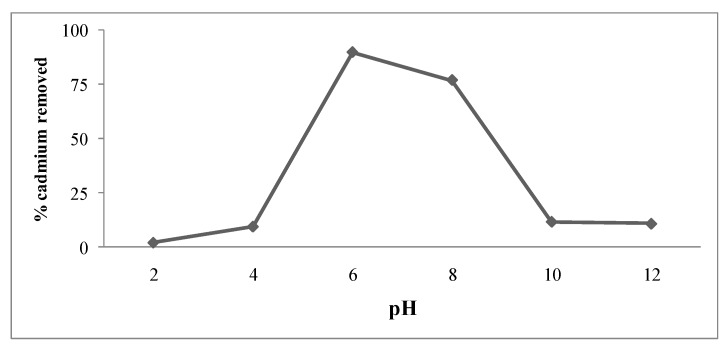
Effect of pH on cadmium removal by *Pseudomonas* sp. strain Al-Dhabi-126.

The pH is one of the important factors that affect the chemical behavior of metal ions in solution and the bacterial metabolic process. The effect of pH on Cd removal by a bacterial strain is presented in Figure 7. In the present findings, Cd removal was found to be higher at a pH between 6.0 and 8.0. A sharp decrease in Cd resistance was noted after pH 9.0 (Figure 7). The variation in the bioremediation process due to pH changes could be due to the difference in the protonation of ligands of *Pseudomonas* on its cell surface. The differences in external pH in the medium can significantly affect the degree of the protonation of ligands that involves metal binding [38]. In *Pseudomonas fluorescens*, Cd removal efficacy was found to be high at pH 6.8 [39]. In a study, optimum pH was reported as 7.0 for bacterial isolates in heavy metal bioremediation [40] and this was higher than the present findings. In the bioremediation process, pH influenced the solubility of the metal ions in the medium, and this process mainly depends on the functional groups of bacterial cell surfaces [41]. In microorganisms, functional groups such as hydroxyl, carboxyl, phosphate, and amino play a major role in the uptake of Cd and this behavior varies at various pH conditions [42]. At lower pH values (less than 4.0) adsorption behavior of the various metal is generally low and significant adsorption behavior is achieved at a medium pH, ranged between 5.0 and 9.0 [43]. In the present study, bioremediation of Cd was found to be at its maximum at pH (6.0–9.0). The negatively charged phosphate, carboxylic groups enhanced the absorption of positive-charged metal ions at higher pH values [44]. In a study, Park et al. [26] reported that the optimum pH value for bioremediation of heavy metal ranges between 5.5 and 6.5, however this pH ranges varied widely.

Henriques and Love [45] reported 80% removal of Cd using *Pseudomonas putida* strain from the culture medium. The cell walls or the envelope of bacteria are able to absorb metal ions from the medium by electrostatic interactions, and the heavy metal removing mechanism is a nonspecific interaction of heavy metals to the extracellular polysaccharides or cell envelope, proteins, teichoic acids, siderophores, and teichronic acids [46]. Extracellular polysaccharides (EPS) are also involved in the bioaccumulation of heavy metals. These EPS contain many functional groups namely amide, carboxyl, imidazole, amino, hydroxyl, phosphate, sulfhydryl, carbonyl, amide, and phosphodiester groups that give very strong negative charge [47]. So metal ions from the medium may be attracted to the cell surface of bacteria. The heavy metals from the environment are also transported through the membrane of the bacteria through the permeation of lipids, carrier-mediated transport, endocytosis, complex permeation, and ion pumps.

Many bacterial genera, including *Pseudomonas* and *Bacillus,* have potential heavy metal removing efficacy [48]. It was previously reported that the outer membrane of the bacteria can absorb more than 30 varieties of metals [49]. Panwichian et al. [18] stated that Pseudomonas aeruginosa strain eliminated more than 75% of the Cd from Cd-amended industrial wastewater under laboratory conditions. Bioremediation of metal pollution of the soil environment has various applications, including being a low-cost procedure for the maintenance of the soil structure. The present finding shows that the isolate Al-Dhabi 126 can be well used for bioremediation.

## 4. Conclusions

The ability of the selected bacterial strain Al-Dhabi 126 to grow in the presence of Cd would be highly useful in the wastewater treatment or bioremediation of toxic xenobiotic components. In this study, *Pseudomonas* strain Al-Dhabi-126, isolated from the metal-contaminated environment, was highly resistant to Cd and had grown well at a dose level of 2100 µg/mL. Al-Dhabi-126 effectively removed Cd at a pH between 6.0 and 8.0. This present study suggests the application of the strain Al-Dhabi-126 for the effective bioremediation of water contaminated with Cd.

## Figures and Tables

**Table 1:** Total viable cell count of bacterial colonies from the heavy metal contaminated sample.

Dilution	Control	Cd-Incorporated	Cd Resistant
Factor		LB Media	Bacteria (%)
10^−1^	13.2 × 10^2^	4.3 × 10^2^	32.5
10^−2^	8.2 × 10^3^	2.1 × 10^3^	25.6
10^−3^	1.2 × 10^4^	0.67 × 10^4^	55.8
10^−4^	0.6 × 10^5^	0.33 × 10^5^	55
10^−5^	0.31 × 10^6^	0.13 × 10^6^	41.9

**Table 2:** Morphological and biochemical characters of the selected bacterial strains for Cd removal.

Tests	Bacterial Strains					
	Al-Dhabi-122	Al-Dhabi-123	Al-Dhabi-124	Al-Dhabi-125	Al-Dhabi-126	Al-Dhabi-127
Gram’s staining	+	+	-	+	+	-
Colony color	White	White milky	Whitish	White milky	White	Yellowish
Shape	Rod	Circle	Round	Rod	Rod	Round
Catalase	+	+	+	+	+	+
Oxidase	-	+	+	-	+	+
Indole	-	-	-	-	-	-
Citrate	-	+	+	-	+	+
Methyl-Red	-	-	-	+	+	-
Sucrose	+	+	-	+	+	-
Glucose	+	-	-	+	+	-
Xylose	+	-	-	-	-	+
Maltose	-	+	-	+	+	+
Lactose	-	+	-	+	-	-
